# Supplementary material for: Vulnerable Factors Affecting Urinary N-Methylformamide Concentration among Migrant Workers in Manufacturing Industries in Comparison with Native Workers in the Republic of Korea (2012–2019)
Source: Int J Environ Res Public Health. 2022 Oct 18;19(20):13450. doi: 10.3390/ijerph192013450 (PMC9602757; doi:10.3390/ijerph192013450)
Supplement: Supplementary file 1 [file ijerph-19-13450-s001.zip › ijerph-1923083-supplementary.pdf]

Supplement Table S1. Subgroup analysis between the native and the migrant workers based on the level of urine concentration of N′-methylformamide

Characteristics	Low and moderate (≤30 mg/L)			High (>30 mg/L)		
Nationality	Native workers (N=9,164)	Migrant workers (N=476)	P[a]	Native workers (N=95)	Migrant workers (N=28)	P[a]
**Sex, n(%)**			0.372			0.099
Male	7555 (82.4)	387 (81.3)		74 (77.9)	26 (92.9)	
Female	1609 (17.6)	89 (18.7)		21 (22.1)	2 (7.1)	
**Age group, n(%)**			<0.001			0.017
20-29 years	2847 (31.1)	201 (42.2)		21 (22.1)	13 (46.4)	
30-39 years	2666 (29.1)	190 (39.9)		15 (15.8)	7 (25.0)	
40-49 years	2016 (22)	61 (12.8)		25 (26.3)	3 (10.7)	
≥50 years	1635 (17.8)	24 (5)		34 (35.8)	5 (17.9)	
**Number of workers, n(%)**			<0.001			0.083
<5	106 (1.2)	20 (4.2)		6 (6.3)	3 (10.7)	
5-50	1643 (17.9)	260 (54.6)		59 (62.1)	22 (78.6)	
≥50	7415 (80.9)	196 (41.2)		30 (31.6)	3 (10.7)	
**Types of industries, Manufactures of , n(%)**			<0.001			0.018
Rubber and plastics products	1025 (11.2)	117 (24.6)		9 (9.5)	8 (28.6)	
Wearing apparel	223 (2.4)	69 (14.5)		7 (7.4)	2 (7.1)	
Textiles	393 (4.3)	52 (10.9)		13 (13.7)	6 (21.4)	
Chemicals and chemical products	3539 (38.6)	58 (12.2)		10 (10.5)	-	
Leather and related products	201 (2.2)	35 (7.4)		35 (36.8)	11 (39.3)	
Other manufacturing	3783 (41.3)	145 (30.5)		21 (22.1)	1 (3.6)	
**TWA of DMF, n(%)[b]**			<0.001			0.284
Low	5442 (59.4)	132 (27.7)		4 (4.2)	2 (7.1)	
Moderate	3194 (34.9)	254 (53.4)		39 (41.1)	7 (25)	
High	528 (5.8)	90 (18.9)		52 (54.7)	19 (67.9)	
**Years of employment, years(SD)**	7.83 (9.04)	1.61(2.08)	<0.001	3.31 (6.05)	1.61(1.71)	0.577

a. P values were calculated using the statistics of Pearson's chi-square and Mann-whitney test

b. The employees having records of ‘0’ or ‘ND ’ were defined as ‘Low’ group. Others were classified into ‘Moderate’ and ‘High' based on 5ppm, the adopted value as TLVs in ACGIH

DMF, Dimethyl Formamide; SD, Standard Deviation; TWA, Time-weighted average; ND, Not detected; TLVs, Threshold limit values; ACGIH, American Conference of Governmental Industrial Hygienists
